# Session Recommendation via Recurrent Neural Networks over Fisher Embedding Vectors †

**DOI:** 10.3390/s19163498

**Published:** 2019-08-10

**Authors:** Domokos Kelen, Bálint Daróczy, Frederick Ayala-Gómez, Anna Ország, András Benczúr

**Affiliations:** 1 Institute for Computer Science and Control, Hungarian Academy of Sciences (MTA SZTAKI), H-1111 Budapest, Hungary; 2 Faculty of Informatics, Eötvös University, Pázmány sétány 1/C, H-1117 Budapest, Hungary; 3 Széchenyi University, Egyetem tér 1, H-9026 Győr, Hungary

**Keywords:** recommender systems, recurrent neural networks, fisher information, markov random fields

## Abstract

Recommendation services bear great importance in e-commerce, shopping, tourism, and social media, as they aid the user in navigating through the items that are most relevant to their needs. In order to build recommender systems, organizations log the item consumption in their user sessions by using different sensors. For instance, Web sites use Web data loggers, museums and shopping centers rely on user in-door positioning systems to register user movement, and Location-Based Social Networks use Global Positioning System for out-door user tracking. Most organizations do not have a detailed history of previous activities or purchases by the user. Hence, in most cases recommenders propose items that are similar to the most recent ones viewed in the current user session. The corresponding task is called session based, and when only the last item is considered, it is referred to as item-to-item recommendation. A natural way of building next-item recommendations relies on item-to-item similarities and item-to-item transitions in the form of “people who viewed this, also viewed” lists. Such methods, however, depend on local information for the given item pairs, which can result in unstable results for items with short transaction history, especially in connection with the cold-start items that recently appeared and had no time yet to accumulate a sufficient number of transactions. In this paper, we give new algorithms by defining a global probabilistic similarity model of all the items based on Random Fields. We give a generative model for the item interactions based on arbitrary distance measures over the items, including explicit, implicit ratings and external metadata to estimate and predict item-to-item transition probabilities. We exploit our new model in two different item similarity algorithms, as well as a feature representation in a recurrent neural network based recommender. Our experiments on various publicly available data sets show that our new model outperforms simple similarity baseline methods and combines well with recent item-to-item and deep learning recommenders under several different performance metrics.

## 1. Introduction

Consider a museum that wants to provide a virtual guide for visitors that explains the items in an exhibition and keeps tracks of the items viewed during the visit with a beacon by using in-door and out-door positioning systems for tracking. Using the list of items viewed, the museum can suggest unseen items that might be relevant [1,2,3]. In such applications, the task is to recommend relevant items to a user based on items seen during the current session [4] rather than on user profiles.

An intuitive approach to building the list of relevant items to recommend in a user session is to compare the attributes of the most recent item against those of candidate next items, and select the most similar one. Such naive methods only use the attributes of the item pair in question. When considering more complex patterns, it becomes challenging to deal with the high-dimensional and nonlinear complete sensor data collection [5]. More data provides more accurate prediction, however at the same time, useful knowledge might be submerged in large amounts of redundant data.

More recent methods construct a notion of similarity based on global information, for example, by dimensionality reduction [6] or by building a neural embedding [7]. Dimensionality reduction is often used to obtain a more compact representation of the original high-dimensional data, a representation that nonetheless captures all the information necessary for higher-level decision-making [8].

Our goal is to use global information for defining item similarity, which can help to handle rare and new items and tackle the so-called cold start case [9] where the new items do not yet have sufficient number of interactions to reliably model their relation to the user. The main difficulty compared to the traditional dimensionality reduction task is that a session is too short to gather meaningful description over the user side of the data, hence dimensionality reduction has to be performed by partial information, the item side of the data only.

Our key idea is to define a notion of similarity based on the global characteristics of the items, possibly combining multiple modes, such as user feedback, content, and metadata. Our starting point is the Euclidean Item Recommender (EIR) method of [6], which utilizes all training data to estimate item-item conditional probabilities through latent factor vectors, which are learned globally.

Our new algorithm is based on a simple generative model for the occurrence and co-occurrence of items. The generative model itself can be defined by combining and augmenting standard similarity measures, such as Jaccard or Cosine based on collaborative, content, multimedia, and metadata information. As a common practice, especially with cold-start items, we also include the item attributes to compute similarities. As an example, in the use case of the person visiting a museum with a in-door positioning device, the recommender system could use the content of the viewed items to improve the recommendations of the next items to visit. We incorporate content similarity in our experiments by mapping the movies in the MovieLens data set to DBpedia [10].

Rather than using our generative model directly for recommendation, we utilize the tangent space of the model to derive mathematically grounded feature representations for each item. We compute an approximation of the Fisher vector corresponding to the Gibbs distribution of the generative model. Our method is based on the theory described in a sequence of papers with the most important steps including [11,12,13], which are in turn used in most of the state-of-the-art image classification methods [14,15].

We propose two direct ways of using the space of Fisher vectors for item to item recommendation. In addition, we also utilize the Fisher vectors by considering them as a predefined embedding in Recurrent Neural Network (RNN) recommender models. The past few years have seen the tremendous success of deep neural networks in several tasks [16]. Sequential data modeling has also recently attracted a lot of attention based on various RNNs [17,18]. In recommender systems, recurrent networks were perhaps first used in the Gru4Rec algorithm [7]. In our best performing algorithm, we replace the dynamically trained neural embedding of Gru4Rec by the precalculated Fisher vectors.

We experimentally show that item-to-item recommendations based on the similarity of the Fisher vectors perform better than both traditional similarity measures and the Euclidean Item Recommender [6]. For session recommendation, by replacing the neural embedding in Gru4Rec [7] with the Fisher vectors, we obtain a class of methods based on different item descriptors that combine well and improve the recommendation quality of Gru4Rec. We evaluate our experiments using top-*n* recommendation [19] performance of our models by MPR [6], Recall, and DCG [20].

Our key contributions in this research can be summarized as follows:

- We propose a novel application of Fisher vectors by using them as item representations in recommender systems. We symbolically derive the Fisher vectors for our tasks and give approximate algorithms to compute them.
- We propose two ways of using the representations for item-to-item recommendation. We measure a performance improvement compared to prior methods.
- We examine the usage of Fisher vectors as a predefined embedding in recurrent neural network based recommendation systems and measure competitive, in some cases even significantly improved, performance compared to dynamically trained neural embedding methods.

The rest of this paper is organized as follows. After the related results, in Section 3 we describe traditional item pair similarity measures and our Fisher vector based machinery for defining similarity based on global item information. In Section 4 we give a brief overview of the Gru4Rec algorithm [7] and show how we can incorporate Fisher vectors by replacing the neural item embeddings. In Section 5 we describe the experimental data sets, settings, and algorithms, and in Section 6 we present our experimental evaluation.

## 2. Related Results

Recommender systems surveyed in [21] have become common in a variety of areas including movies, music, tourism, videos, news, books, and products in general. They produce a list of recommended items by either collaborative or content-based filtering. Collaborative filtering methods [4,22,23] build models of past user-item interactions, while content-based filtering [24] typically generates lists of similar items based on item properties. Recommender systems rely on explicit user feedback (e.g., ratings, likes, dislikes) or implicit feedback (e.g., clicks, plays, views) to assess the attitude towards the items viewed by the user.

The Netflix Prize Challenge [25,26] has revolutionized our knowledge of recommender systems, but caused bias in research towards scenarios where user profiles and item ratings (1–5 stars) are known. However, for most Web applications, users are reluctant to create logins and prefer to browse anonymously. In other cases, users purchase certain types of goods (e.g., expensive electronics) so rarely that previous purchases are insufficient to create a meaningful user profile. Several practitioners [6] argue that most of the recommendation tasks they face count as implicit feedback and are without sufficient user history. In [27] the authors claim that 99% of the recommendation systems they built for industrial application tasks are implicit, and most of them are item-to-item. For these cases, recommender systems rely on the recent items viewed by the user in the given shopping session.

The first item-to-item recommender methods [4,22] used similarity information to find nearest neighbor transactions [28]. Another solution is to extract association rules [29]. The method outlined in [30] learns similarity weights for users; however, the method gives global and not session-based user recommendation.

Rendle et al. [31] proposed a session-based recommender system that models the users by factorizing personal Markov chains. Their method is orthogonal to ours in that they provide more accurate user-based models if more data is available, while we concentrate on extracting actionable knowledge from the entire data set for the sparse transactions in a session.

Item-to-item recommendation can be considered a particular context-aware recommendation problem. In [32] sequentiality as a context is handled by using pairwise associations as features in an Alternating Least Squares (ALS) model. The authors mention that they face the sparsity problem in setting minimum support, confidence, and lift of the associations, and they use the category of last purchased item as a fallback. In a follow-up result [33], they use the same context-aware ALS algorithm. However, they only consider seasonality as a context in the latter paper.

In case of sequential item-to-item recommendation, we exploit our knowledge about previous item transitions. The closest to our work is the *Euclidean Item Recommender* [6] by Koenigstein and Koren. They model item-to-item transitions using item latent factors where the Euclidean distance between two vectors approximates the known transition probabilities in the training data set. Our model differs in that we do not need to optimize a vector space to learn the transition probabilities in a lower dimensional space. Instead, we start from an arbitrary similarity definition, and we can extend similarity for all items by using all training data, in a mathematically justified way. We use Fisher information that has been applied for DNA splice site classification [12] and computer vision [13], but we are the first to apply it in recommender systems. In our experiment, we made an effort to reproduce the experimental settings of EIR to the greatest extent possible.

Recurrent Neural Networks have been applied in capturing temporal dynamics of implicit and explicit recommendation. One of the first uses of neural networks for recommendation is the Restricted Boltzmann Machines (RBM) method for Collaborative Filtering [34]. In this work, an RBM is used to model user-item interaction and perform recommendations. Hochreiter in [35] showed that simple recurrent units are not entirely sufficient to describe long-term dependencies, and together with Schmidhuber he suggested Long-Short Term Memory (LSTM) in [18]. Cho et al. [17] proposed a less complex recurrent unit, the Gated Recurrent Unit (GRU). Hidasi et al. [7] built a widely used neural network structure, the Gru4Rec with GRU and a specific input, output embedding for sequential recommendation. Their model transforms a high dimensional one-hot coded item representation into a relative small dimensional but dense embedded vector. The context-free embedding vectors act as input to the single GRU layer with output gates, and they are finally transformed back into the high dimensional, itemset-sized probabilistic space. During training, the model is optimized for predicting the next item in the sequence.

Finally, we review the results of extending content description by knowledge graphs. To help with the cold-start problem, it is a common practice to include the attributes of the items in the recommender system. For example, in the case of recommender system based on a in-door positioning device, we could use the content of the viewed items to improve the recommendations of additional items [36,37].

Knowledge-based recommendation systems include the characteristics of the required item [38]. The characteristics of items and their descriptions is crucial for a knowledge-based recommendation system to make accurate recommendations [39]. Knowledge about items can be compiled as statements, rules or ontologies [40] using case-based or rule-based reasoning [41] such that knowledge is extracted from a case database [42].

Linked open data has been used in several results to support content-based recommender systems [43], our main result is the fusion of such techniques with collaborative filtering. The main example of linked open data is DBPedia [10], a popular ontology used in recommender systems [44]. Such systems are used for recommender systems in several domains, including music [45] and tourism [46]. However, the methods to fuse similarity based on ontologies and other techniques do not go beyond simple score combination by using stacking [47].

## 3. Item-to-Item Similarity Measures

The natural way of item-to-item recommendation is to rank the next candidate items based on their similarity to the last visited item. While the idea is simple, a wide variety of methods exist to compute the distance or divergence of user feedback, content, and other potential item metadata.

In this section, first we enumerate the most common similarity measures among raw item attributes, which will also serve as the baseline in our experiments. Then we describe our new methodology that introduces an item representation in a kernel space built on top of the raw similarity values. Based on the item representation, we define three different types of “kernelized”, transformed similarity measures.

### 3.1. Raw Attribute Similarity Measures

First, we enumerate distance and divergence measures that directly compare the attributes of the item pair in question. We list both implicit feedback collaborative filtering and content-based measures. The raw attribute similarity formulas yield the natural baseline methods for item-to-item recommendation.

For user implicit feedback on item pairs, various joint and conditional distribution measures can be defined based on the frequencies $f_{i}$ and $f_{ij}$ of items *i* and item pairs i,j, as follows.

1. Cosine similarity (Cos):
  (1) $$cos\left(i,j\right)=\frac{f_{ij}}{\sqrt{f_{i}f_{j}}}.$$
2. Jaccard similarity (JC):
  (2) $$JC\left(i,j\right)=\frac{f_{ij}}{f_{i}+f_{j}-f_{ij}}.$$
3. Empirical Conditional Probability (ECP), which estimates the item transition probability:
  (3) $$ECP\left(j|i\right)=\frac{f_{ij}}{f_{i}+1},$$
  where the value 1 in the denominator is used for smoothing.

Additionally, in [6] the authors suggest the Euclidean Item Recommender (EIR) model to approximate the transition probabilities with the following conditional probability

(4) $$p\left(j|i\right)=\frac{\exp^{-\left|\right|x_{i}-x_{j}{\left|\right|}^{2}+b_{j}}}{\sum_{k\in T}\exp^{-\left|\right|x_{i}-x_{k}{\left|\right|}^{2}+b_{k}}},$$

where *T* is the set of items. They learn the item latent vectors $\left\{x_{i}\right\}$ and biases $\left\{b_{i}\right\}$.

Besides item transitions, one can measure the similarity of the items based on their content (e.g., metadata, text, title). We will measure the content similarity between two items by the Cosine, Jaccard, tf-idf, and the Jensen–Shannon divergence of the bag of words representation of the metadata description.

### 3.2. Similarity in the DBPedia Knowledge Graph

We obtain text description of MovieLens movies by mapping them to DBpedia (http://wiki.dbpedia.org) [10]. DBpedia is the representation of Wikipedia as a knowledge graph described in the machine readable Resource Description Framework (RDF). RDFs are triplets of the resource, the property, and the property value, all identified by a Uniform Resource Identifier (URI), which we use for defining the item description vocabulary.

We compute the Jaccard similarity between two items using the nodes connected to the movies represented by their graphs. For an item *i*, we build the set of properties $i_{\mathrm{prop}}$ of the neighboring resources. The Jaccard similarity between the items is defined by the formula

(5) $$sim\left(i,j\right)=\frac{|i_{\mathrm{prop}}⋂j_{\mathrm{prop}}|}{|i_{\mathrm{prop}}⋃j_{\mathrm{prop}}|}.$$

### 3.3. Notion of Similarity Based on Fisher Information

In this section, we describe our new method, a feature representation of the items that augments the similarity measures of the previous section by using global information. Our representation can be the basis of performance improvement, since it relies on global structural properties rather than simple statistics between the user feedback or content of the two items in question. In addition, by starting out with multimodal similarity measures, including implicit or explicit user feedback, user independent metadata, such as text description, linkage, or even multimedia content, our machinery yields a parameter-free combination of the different item descriptions. Hence, the current section builds upon and fuses all the possible representations described in the previous sections.

Our method is based on the theory of the tangent space representation of items described in a sequence of papers with the most important steps including [11,12,13]. In this section, we describe the theory and its adaptation to the item-to-item recommendation task where we process two representations, one for the previous and one for the current item.

After describing our feature representation, in the subsequent subsections we give three different distance metrics in the representing space, based on different versions of the feature representations. We start our definition of global structural similarity by considering a set of arbitrary item pair similarity measures, such as the ones listed in the previous section. We include other free model parameters θ, which can, for example, serve to scale the different raw attributes or the importance of attribute classes. We give a generative model of items *i* as a random variable p(i|θ). From p(i|θ), we infer the distance and the conditional probability of pairs of items *i* and *j* by using all information in θ.

**Figure 1:**
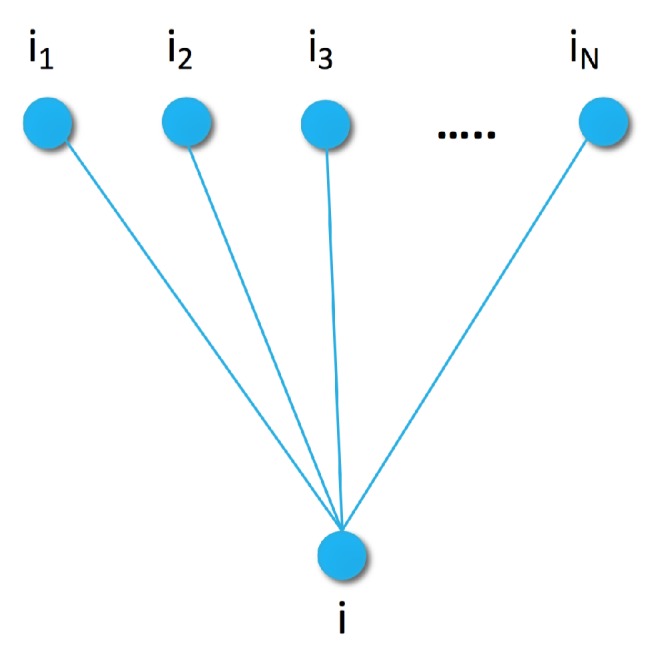
Similarity graph of item *i* with sample items $S=\{i_{1},i_{2},...,i_{N}\}$ of distances $\mathrm{dist}(i,i_{n})$ from *i*.

To define the item similarity generative model, let us consider a certain sample of items $S=\{i_{1},i_{2},\ldots ,i_{N}\}$ (e.g., most popular or recent items), and assume that we can compute the distance of any item *i* from each of $i_{n}\in S$. We consider our current item *i* along with its distance from each $i_{n}\in S$ as a random variable generated by a Markov Random Field (MRF). Random Fields are a set of (dependent) random variables. In case of MRF, the connection between the elements is described by an undirected graph satisfying the Markov property [48]. For example, the simplest Markov Random Field can be obtained by using a graph with edges between item *i* and items $i_{n}\in S$, as shown in Figure 1.

Let us assume that we are given a Markov Random Field generative model for p(i|θ). By the Hammersley–Clifford theorem [49], the distribution of p(i|θ) is a Gibbs distribution, which can be factorized over the maximal cliques and expressed by a potential function *U* over the maximal cliques as follows:

(6) $$p\left(i|\theta \right)=e^{-U(i|\theta )}/Z(\theta ),$$

where U(i∣θ) is the energy function and

(7) $$Z\left(\theta \right)=\sum_{i}e^{-U(i|\theta )}$$

is the sum of the exponent of the energy function over our generative model, a normalization term called the partition function. If the model parameters are previously determined, then Z(θ) is a constant.

By using the Markov Random Field defined by the graph in Figure 1, or some more complex ones defined later, a wide variety of proper energy functions can be used to define a Gibbs distribution. The weak but necessary restrictions are that the energy function has to be positive real valued, additive over the maximal cliques of the graph, and more probable parameter configurations have to have lower energy.

We define our first energy function for (6) based on the similarity graph of Figure 1. Since the maximal cliques of that graph are its edges, the energy function has the form

(8) $$U\left(i|\theta =\left\{\alpha_{1},..,\alpha_{N}\right\}\right):=\sum_{n=1}^{N}\alpha_{n}\mathrm{dist}\left(i,i_{n}\right),$$

where $S=\{i_{1},..,i_{N}\}$ is a finite sample set, dist is an arbitrary distance or divergence function of item pairs, and the hyperparameter set θ is the weight of the elements in the sample set.

**Figure 2:**
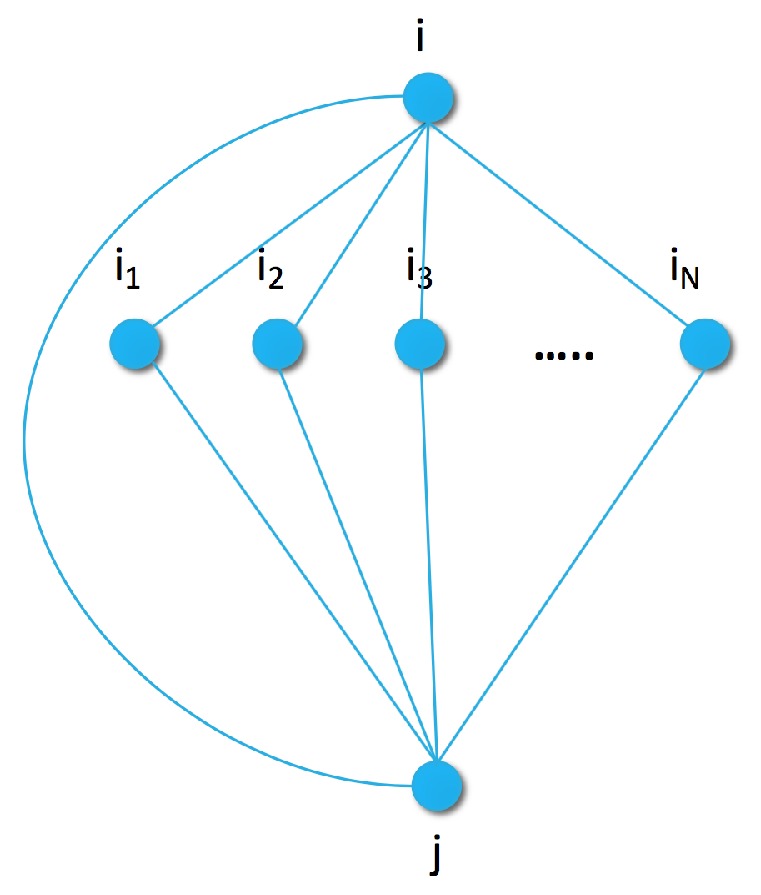
Pairwise similarity graph with sample set $S=\{i_{1},i_{2},...,i_{N}\}$ for a pair of items *i* and *j*.

In a more complex model, we capture the connection between pairs of items by extending the generative graph model with an additional node for the previous item as shown in Figure 2. In the pairwise similarity graph, the maximal clique size increases to three. To capture the joint energy with parameters $\theta =\{\beta_{n}\}$, we can use a heuristic approximation similar to the pseudo-likelihood method [48]: we approximate the joint distribution of each size three clique as the sum of the individual edges by

(9) $$U\left(i,j|\theta \right):=\sum_{n=1}^{N}\beta_{n}\left(\mathrm{dist}\left(i,i_{n}\right)+\mathrm{dist}\left(j,i_{n}\right)+\mathrm{dist}\left(i,j\right)\right).$$

At first glance, the additive approximation seems to oversimplify the clique potential and falls back to the form of Equation (8). However, the three edges of clique *n* share the hyperparameter $\beta_{n}$, which connects these edges in our modeling approach.

Based on either of the energy functions in Equation (8) or (9), we are ready to introduce the Fisher information to estimate distinguishing properties by using the similarity graphs. Let us consider a general parametric class of probability models p(i|θ) where $\theta \in \Theta \subseteq R^{\ell }$. The collection of models with parameters from a general hyperparameter space Θ can then be viewed as a (statistical) manifold $M_{\Theta }$, provided that the dependence of the potential on Θ is sufficiently smooth. By [50], $M_{\Theta }$ can be turned into a Riemann manifold by giving an inner product (kernel) at the tangent space of each point $p\left(i|\theta \right)\in M_{\Theta }$ where the inner product varies smoothly with *p*.

The notion of the inner product over p(i|θ) allows us to define the so-called Fisher metric on *M*. The fundamental result of Čencov [11] states that the Fisher metric exhibits a unique invariance property under some maps, which are quite natural in the context of probability. Thus, one can view the use of Fisher kernel as an attempt to introduce a natural comparison of the items on the basis of the generative model [12].

We start by defining the Fisher kernel over the manifold $M_{\Theta }$ of probabilities p(i|θ) as in Equation (6), by considering the tangent space. The tangent vector, which is the row vector defined as

(10) $$G_{i}=\nabla {\;}_{\theta }\log p\left(i|\theta \right)=\left(\frac{\partial }{\partial \theta_{1}}\log p\left(i|\theta \right),\ldots ,\frac{\partial }{\partial \theta_{N}}\log p\left(i|\theta \right)\right),$$

is called the *Fisher score* of (the occurrence of) item *i*. An intuitive interpretation is that $G_{i}$ gives the direction where the parameter vector θ should be changed to fit item *i* the best [13]. The *Fisher information matrix* is a positive definite matrix of size N×N, defined as

(11) $$F\left(\theta \right):=E_{\theta }\left(\nabla {\;}_{\theta }\log{p(i|\theta )}^{T}\;\nabla {\;}_{\theta }\log p(i|\theta )\right),$$

where the expectation is taken over p(i|θ), i.e.,

$$F{\left(\theta \right)}_{nm}=\sum_{i\in T}p\left(i|\theta \right)\left(\frac{\partial }{\partial \theta_{n}}\log p(i|\theta )\right)\left(\frac{\partial }{\partial \theta_{m}}\log p(i|\theta )\right),$$

where *T* is the set of all items. The corresponding kernel function

(12) $$K\left(i,j\right):=G_{i}F^{-1}G_{j}^{T}$$

is called the *Fisher kernel*. We further define the *Fisher vector* of item *i* as

(13) $$G_{i}=G_{i}F^{-\frac{1}{2}},$$

so that the equation

(14) $$G_{i}G_{j}^{T}=K\left(i,j\right)$$

holds (as *F* is symmetric).

Thus, to capture the generative process, the gradient space of $M_{\Theta }$ is used to derive the Fisher vector, a mathematically grounded feature representation of item *i*.

### 3.4. Item-to-Item Fisher Distance (FD)

Based on the feature representation framework of the previous section, in the next three subsections we propose three item similarity measures.

Our first measure arises as an inner product of the Fisher vectors. Any inner product can be used to obtain a metric by having $∥u∥={\langle u,u\rangle }^{\frac{1}{2}}$. Using the Fisher kernel K(i,j), the *Fisher distance* can be formulated as

(15) $${\mathrm{dist}}_{F}\left(i,j\right)={∥G_{i}-G_{j}∥}_{K}=\sqrt{K(i,i)-2K(i,j)+K(j,j)}.$$

Thus, we need to compute the Fisher kernel over our generative model as in (12). By substituting into (15), the recommended next item after item *i* will be

(16) $$j^{*}=\underset{j\neq i}{\text{arg min}}{\mathrm{dist}}_{F}\left(i,j\right).$$

The computational complexity of the Fisher information matrix estimated on the training set is $O(T|\theta {|}^{2})$ where *T* is the size of the training set. To reduce the complexity to O(T|θ|), we can approximate the Fisher information matrix with the diagonal as suggested in [12,13]. Our aim is then to compute

$$G_{i}=G_{i}F^{-\frac{1}{2}}\approx G_{i}F_{\mathrm{diag}}^{-\frac{1}{2}}.$$

For this, we observe that

(17) $$\begin{array}{cc} G_{i}^{k}\left(\theta \right) & =\frac{\partial }{\partial \theta_{k}}\log p(i|\theta )=\frac{\partial }{\partial \theta_{k}}\log\frac{e^{-U(i|\theta )}}{\sum_{j\in S}e^{-U(j|\theta )}}\\ & =\sum_{l\in T}\frac{e^{-U(j|\theta )}}{\sum_{j\in S}e^{-U(j|\theta )}}\frac{\partial U(l|\theta )}{\partial \theta_{k}}-\frac{\partial U(i|\theta )}{\partial \theta_{k}}\\ & =\sum_{l\in T}p\left(l|\theta \right)\frac{\partial U(l|\theta )}{\partial \theta_{k}}-\frac{\partial U(i|\theta )}{\partial \theta_{k}}, \end{array}$$

and also that

(18) $$\frac{\partial U(i|\theta )}{\partial \theta_{k}}=\mathrm{dist}\;\left(i,i_{k}\right).$$

Combining (17) and (18), we get

(19) $$G_{i}^{k}\left(\theta \right)=E_{\theta }\;\left(\mathrm{dist}\;\left(i,i_{k}\right)\right)-\mathrm{dist}\;\left(i,i_{k}\right).$$

Also, since

(20) $$F_{kk}=E_{\theta }\left[{\left(\frac{\partial }{\partial \theta_{k}}\log p(i|\theta )\right)}^{2}\right],$$

by (17) and (18)

(21) $$F_{kk}=E_{\theta }\left[{\left(E_{\theta }\;\left(\mathrm{dist}\;\left(i,i_{k}\right)\right)-\mathrm{dist}\;\left(i,i_{k}\right)\right)}^{2}\right],$$

i.e., for the energy functions of Equations (8) and (9), the diagonal of the Fisher kernel is the standard deviation of the distances from the samples.

Finally, using this information, we are able to compute $G_{i}$ as

(22) $$G_{i}^{k}=\frac{E_{\theta }\;\left[\mathrm{dist}\;\left(i,i_{k}\right)\right]-\mathrm{dist}\;\left(i,i_{k}\right)}{E_{\theta }^{\frac{1}{2}}\left[{\left(E_{\theta }\;\left(\mathrm{dist}\;\left(i,i_{k}\right)\right)-\mathrm{dist}\;\left(i,i_{k}\right)\right)}^{2}\right]},$$

which gives us the final kernel function as

(23) $$\begin{array}{cc} K(i,j) & =G_{i}F^{-1}G_{j}^{T}\approx G_{i}F_{diag}^{-1}G_{j}^{T}\\ & =G_{i}F_{diag}^{-\frac{1}{2}}F_{diag}^{-\frac{1}{2}}G_{j}^{T}=\sum_{k}G_{i}^{k}G_{j}^{k}. \end{array}$$

The formula in (22) involves the distance values on the right side, which are readily available, and the expected values on the left side, which can be estimated by using the training data. We note that here we make a heuristic approximation: instead of computing the expected values (e.g., by simulation), we substitute the mean of the distances from the training data.

All of the measures in Section 3 can be used in the energy function as the distance measure after small modifications. Now, let us assume that our similarity graph (Figure 1) has only one sample element *i*, and the conditional item is also *i*. The Fisher kernel will be

(24) $$\begin{array}{cc} K(i,j) & =\frac{1}{\sigma_{i}^{2}}\left(\mu_{i}-\mathrm{dist}\left(i,i\right)\right)\left(\mu_{i}-\mathrm{dist}\left(i,j\right)\right)\\ & =\frac{\mu_{i}^{2}}{\sigma_{i}^{2}}-\frac{\mu_{i}}{\sigma_{i}^{2}}\mathrm{dist}\left(i,j\right)\\ & =C_{1}-C_{2}*\mathrm{dist}\left(i,j\right), \end{array}$$

where $\mu_{i}$ and $\sigma_{i}$ are the expected value and variance of distance from item *i*. Therefore, if we fix θ, $C_{1}$ and $C_{2}$ are positive constants, and the minimum of the Fisher distance is

(25) $$\begin{array}{cc} \min_{j\neq i}{\mathrm{dist}}_{F}\left(i,j\right) & =\min_{j\neq i}\sqrt{K(i,i)-2K(i,j)+K(j,j)}\\ & =\min_{j\neq i}\sqrt{2C_{2}*\mathrm{dist}\left(i,j\right)}=\min_{j\neq i}\mathrm{dist}\left(i,j\right). \end{array}$$

Hence, if we measure the distance over the latent factors of EIR, the recommended items will be the same as defined by EIR, see Equation (10) in [6].

### 3.5. Item-to-Item Fisher Conditional Score (FC)

Our second item similarity measure relies on the item-item transition conditional probability $G_{j|i}\left(\theta \right)$ computed from the Fisher scores of Equation (10). As the gradient corresponds to how well the model fits the sample, the easiest fit as next item $j^{*}$ has the lowest norm; hence,

(26) $$j^{*}=\underset{j\neq i}{\text{arg min}}\left|\right|G_{j|i}\left(\theta \right)\left|\right|.$$

We compute $G_{j|i}\left(\theta \right)$ by the Bayes theorem as

(27) $$\begin{array}{cc} G_{j|i}= & \nabla {\;}_{\theta }\log p(j|i;\theta )=\nabla {\;}_{\theta }\log\frac{p(i,j|\theta )}{p(i|\theta )}=\\ = & \nabla {\;}_{\theta }\log p(i,j|\theta )-\nabla {\;}_{\theta }\log p(i|\theta )=\\ = & G_{ij}-G_{i}. \end{array}$$

To compute, we need to determine the joint and the marginal distributions $G_{ij}$ and $G_{i}$ for a particular item pair. For an energy function as in Equation (8), we have seen in (19) that the Fisher score of *i* has a simple form,

(28) $$G_{i}^{k}\left(\theta \right)=E_{\theta }\left[\mathrm{dist}\left(i,i_{k}\right)\right]-\mathrm{dist}\left(i,i_{k}\right),$$

and it can be seen similarly for Equation (9) that

(29) $$\begin{array}{c} G_{ij}^{k}\left(\theta \right)=E_{\theta }\left[\mathrm{dist}\left(i,i_{k}\right)+\mathrm{dist}\left(j,i_{k}\right)+\mathrm{dist}\left(i,j\right)\right]\\ -(\mathrm{dist}\left(i,i_{k}\right)+\mathrm{dist}\left(j,i_{k}\right)+\mathrm{dist}\left(i,j\right)). \end{array}$$

Now, if we put (28) and (29) into (27), several terms cancel out and the Fisher score becomes

(30) $$G_{j|i}^{k}=E_{\theta }\left[\mathrm{dist}\left(j,i_{k}\right)+\mathrm{dist}\left(i,j\right)\right]-\left(\mathrm{dist}\left(j,i_{k}\right)+\mathrm{dist}\left(i,j\right)\right).$$

Substituting the mean instead of computing the expected value as in Section 3.4, the probabilities $p\left(k,l|\theta \right)=\frac{1}{n^{2}}$. Using this information, we can simplify the above formula:

(31) $$\begin{array}{c} \sum_{r\in T}\sum_{l\in T}\frac{1}{n^{2}}\left[\mathrm{dist}\;\left(l,i_{k}\right)+\mathrm{dist}\;\left(r,l\right)\right]-\left[\mathrm{dist}\;\left(j,i_{k}\right)+\mathrm{dist}\;\left(i,j\right)\right]= \end{array}$$

(32) $$\begin{array}{cc} =\; & \frac{1}{n}\;\sum_{l\in T}\mathrm{dist}\;\left(l,i_{k}\right)+\frac{1}{n^{2}}\;\sum_{r\in T}\sum_{l\in T}\mathrm{dist}\;\left(r,l\right)-\mathrm{dist}\;\left(j,i_{k}\right)-\mathrm{dist}\;\left(i,j\right). \end{array}$$

Since the second term is independent of *k*, it has to be calculated only once, making the computation $O(|T{|}^{2}+|T|N)$. Thus, this method is less computationally efficient than the previous one.

### 3.6. Multimodal Fisher Score and Distance

**Figure 3:**
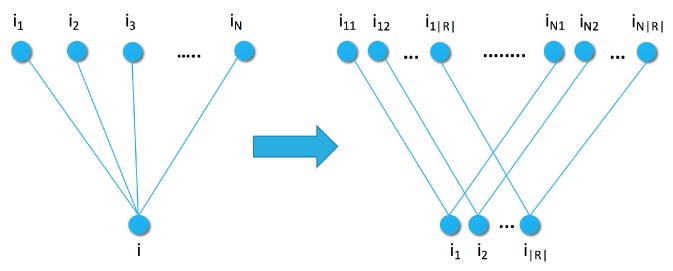
Single and multimodal similarity graph with sample set $S=\{i_{1},i_{2},...,i_{N}\}$ and |R| modalities.

So far we have considered only a single distance or divergence measure over the items. We can expand the model with additional distances with a simple modification to the graph of Figure 1. We expand the points of the original graph into new points $R_{i}=\left\{r_{i,1},..,r_{i,|R|}\right\}$ corresponding to *R* representatives for each item $i_{n}$ in Figure 3. There is an edge between two item representations $r_{i,\ell }$ and $r_{j,k}$ if they are the same type of representation (ℓ=k) and the two items were connected in the original graph. This transformation does not affect the maximal clique size and, therefore, the energy function is a simple addition, as

(33) $$U\left(i|\theta \right)=\sum_{n=1}^{N}\sum_{r=1}^{\left|R\right|}\alpha_{nr}{\mathrm{dist}}_{r}\left(i_{r},i_{nr}\right).$$

If we expand the joint similarity graph to a multimodal graph, the energy function will be

(34) $$\begin{array}{c} U\left(i,j|\theta \right)=\sum_{n=1}^{N}\sum_{r=1}^{\left|R\right|}\beta_{nr}({\mathrm{dist}}_{r}\left(i_{r},i_{nr}\right)\\ +{\mathrm{dist}}_{r}\left(j_{r},i_{nr}\right)+{\mathrm{dist}}_{r}\left(i_{r},j_{r}\right)). \end{array}$$

Now, let the Fisher score for any distance measure r∈R be $G_{ir}$. In that case, the Fisher score for the multimodal graph is the concatenation of the unimodal Fisher scores, as

(35) $$G_{i}^{multi}=\left\{G_{i1},..,G_{i|R|}\right\},$$

and, therefore, the norm of the multimodal Fisher score is a simple sum over the norms:

(36) $$\left|\right|G_{i}^{multi}\left|\right|=\sum_{r=1}^{\left|R\right|}\left|\right|G_{ir}\left|\right|.$$

The calculation is similar for the Fisher kernel of Equation (23); thus, the multimodal kernel can be expressed as

(37) $$K_{multi}\left(i,j\right)=\sum_{r=1}^{\left|R\right|}K_{r}\left(i,j\right).$$

## 4. Recurrent Neural Networks and Fisher Embedding

All similarity measures of Section 3, both the traditional and the Fisher information based ones, utilize only the last consumed item for prediction. Clearly, additional information can be gained from previous items and item transitions in the session. In this section, we give a new method that utilizes the representations of potentially all previous items in the session by incorporating them as an item embedding in a recurrent neural network.

A possible method for predicting the next item based on a sequence of items is Gru4Rec [7], a Recurrent Neural Network model. Gru4Rec transforms a high-dimensional one-hot encoded item representation into a relative small dimensional but dense embedded vector. The model dynamically learns vector embeddings for the items and feeds the representations of the item sequence to GRU units [17] in a neural network. The prediction of the next item is done by a softmax layer, which represents the predicted probability distribution of the next item. During training, the model is optimized for predicting the next item in the sequence.

**Figure 4:**
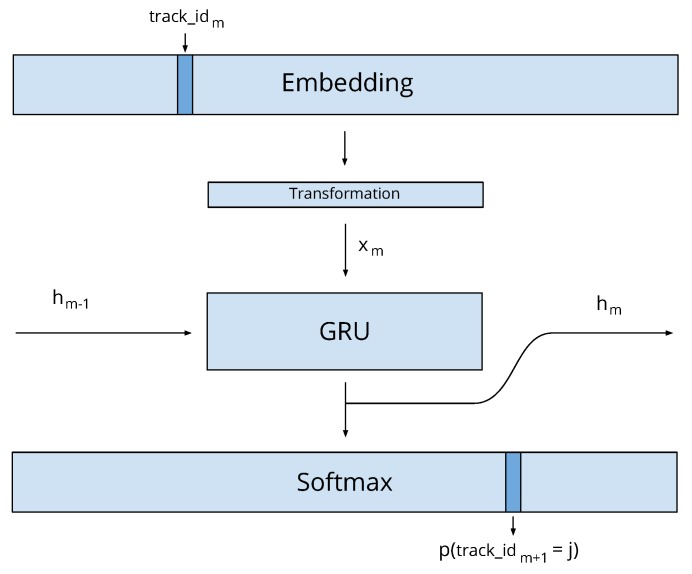
Expanded Gru4Rec model for Fisher embedding.

The Gru4Rec session recommender algorithm reads a sequence of items $i_{1},i_{2},\ldots ,i_{m}$ consumed by a user, and predicts the next item $i_{m+1}$ in the session by estimating the probability distribution $p(i_{m+1}|i_{m},i_{m-1},\ldots ,i_{1})$. Gru4Rec uses GRU units as shown in Figure 4. In each time step *m*, a GRU unit reads an input $x_{m}$ and updates its internal state $h_{m}$:

(38) $$\begin{array}{cc} z_{m} & =\sigma \left(W_{z}x_{m}+U_{z}h_{m-1}\right) \end{array}$$

(39) $$\begin{array}{cc} r_{m} & =\sigma \left(W_{r}x_{m}+U_{r}h_{m-1}\right) \end{array}$$

(40) $$\begin{array}{cc} {\hat{h}}_{m} & =\tanh\left(Wx_{m}+U\left(r_{m}⊙h_{m-1}\right)\right) \end{array}$$

(41) $$\begin{array}{cc} h_{m} & =\left(1-z_{m}\right)h_{m-1}+z_{m}{\hat{h}}_{m}, \end{array}$$

where the matrices *W* and *U* are trainable parameters of the GRU unit.

In our case, $x_{m}=E_{i_{m}}$ is an embedding $E\in R^{|T|\times k}$ of item $i_{m}$ where *T* is the set of items and *k* is the predefined dimensionality of the embedding. Gru4Rec defines another matrix $S\in R^{k\times |T|}$ as the output item representation in the softmax layer that selects the most probable next item to recommend. The model recursively calculates the prediction the following way:

(42) $$\begin{array}{cc} h_{m} & =\mathrm{GRU}(h_{m-1},E_{i_{m}}); \end{array}$$

(43) $$\begin{array}{cc} p(i_{m+1} & =j)=\frac{e^{-h_{m}S_{j}}}{\sum_{n\in T}e^{-h_{m}S_{n}}}. \end{array}$$

Since the matrices *E* and *S* both contain representations of the items, the model can also be defined so that it shares the same parameters for both, i.e., it has the constraint $S=E^{T}$.

In the original Gru4Rec algorithm, matrices *E* and *S* are updated by backpropagation, using the error defined at the output softmax layer. In our modified algorithm, we propose two ways to take advantage of the similarity graphs and Gru4Rec:

1. Instead of using the embedding that we obtain by training the network, we use the Fisher normalized vector from Equation (13).
2. Optionally, we further extend the model with a linear layer, represented by matrix $M\in R^{k\times k}$ and calculate $h_{m}=\mathrm{GRU}\left(h_{m-1},E_{i_{k}}M\right)$, see Figure 4.

The linear transformation *M* is meaningless in the original model; however, it is useful when using the model with precalculated item representations based on Fisher score. In particular, the linear space in Equation (23) can formulate a linear embedding as

$$G_{i}\approx G_{i}F_{\mathrm{diag}}^{-\frac{1}{2}},$$

and the additional quadratic transformation seems to make the diagonal approximation unnecessary, as for a given *i* item the *k*-th element in the transformed embedding will be

$$E_{ik}={\left(G_{i}F_{\mathrm{diag}}^{-\frac{1}{2}}M\right)}_{k}=\sum_{j}G_{ij}f_{j}M_{jk}.$$

Since we use the diagonal approximation $G_{ik}=G_{ik}f_{k}$ of ${\left(G_{i}F^{-1/2}\right)}_{k}$ in the formula, we simply scale the dimensions of the vector $G_{i}$ by constants, which is seemingly made unnecessary by the learned transformation matrix. However, since the optimization process is highly non-convex, we may converge to a completely different suboptimal local optimum without using the scaling provided by the *F*-term.

## 5. Experiments

We performed experiments on four publicly available data sets: Netflix [25], MovieLens (http://grouplens.org/datasets/movielens/), Ziegler’s Books [51], and Yahoo! Music [52]. We randomly split users into a training and a testing set. The number of training and testing pairs and the properties of the data sets can be seen in Table 1.

**Figure 5:**
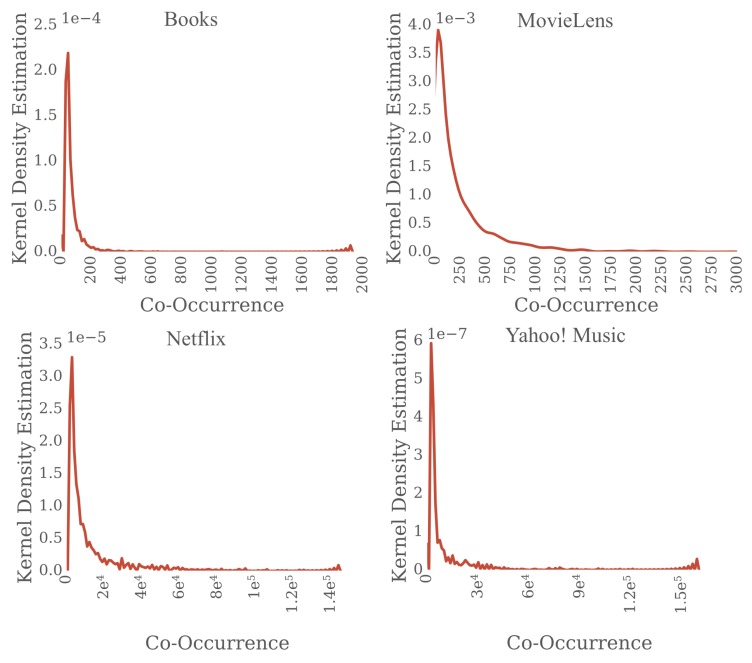
The Kernel Density Estimation function of the item co-occurrence concentrates at infrequent values.

We compute an item transition matrix from the items consumed by the users in sessions of the training data. For example, for a session of items *a*, *b*, and *c*, we create three co-occurrence pairs [(a,b),(b,c),(c,a)]. In a co-occurrence pair (i,j) we call the first element *current item* and the second element *next item*. For each session in the dataset we first generate the co-occurrence pairs, and then calculate the frequency of the pairs and items. Table 2 shows that most co-occurrence pairs in the data sets are infrequent, and 75% of the pairs have low support. Another way to show that most of the pairs are infrequent is to compute the kernel density estimation (KDE) of the frequency of the pairs. KDE [53] is a non-parametric approach for density estimation. Figure 5 shows the KDE plots for the data sets. We observe that most of the co-occurring pairs are infrequent for all the data sets. In our experiments, we focus on infrequent item transitions using only the pairs of items where the *current item* appears with low support in the dataset (i.e., under the 75% percentile). The maximum item support that we considered for the data sets in our experiments is 2 for Books, 23 for Yahoo! Music, 300 for MovieLens and 1241 for Netflix.

**Figure 6:**
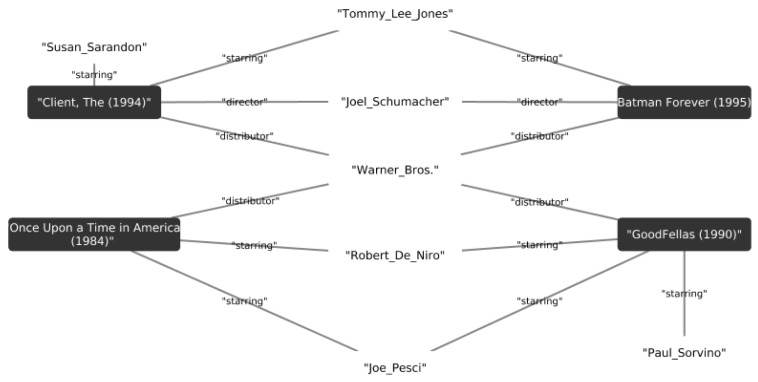
An example of movies from the MovieLens dataset that shows the relations of the movies using the DBpedia knowledge graphs. The black squares show the movie title, the edges are the properties and the white nodes are the property values.

We extended the item metadata of the MovieLens dataset by mapping attributes to DBpedia. By doing this, we enriched the attributes of the movies by connecting them with edges labeled by the director, actors, or genre. Figure 6 presents an example of the relations between movies using some of the properties of DBpedia. We compute the Jaccard similarity between two items using the nodes connected to the movies represented by their graphs.

Out of the 3600 movies in the MovieLens dataset, we were able to map 3100 movies to DBpedia using DBpedia Spotlight [54]. The resulting mapping consists of 371 properties and 146,000 property values. Most properties appear only a few times, as presented in Table 3. Due to sparsity, we only used the some of the properties including starring, writer, genre, director, and producer. Table 4 shows the the most popular values for each of the selected movie properties. We consider the similarity between the movies as the Jaccard similarity of the sets of the corresponding movie properties. Table 5 presents statistics for the Jaccard similarity between the movies using the 100 most similar movies for each item.

As baseline methods, we computed four item-item similarity measures: Empirical Conditional Probability (ECP), Cosine (Cos), and Jaccard as defined in Section 3, and we also implemented the Euclidean Item Recommender of [6] and modified the original Gru4Rec implementation for Fisher embeddings (Code is available at https://github.com/daroczyb/Fisher_embedding). For evaluation, we used MPR [6], Recall, and Discounted Cumulative Gain (DCG) [55].

By following the evaluation method of [6], we sampled 200 random items for each item in the testing set. Given the current item *i* and next item *j* in a session, we used our algorithms to rank *j* along with the random 200 items; we broke ties at random. In our settings, the better the model, the higher the rank of the actual next item *j*.

## 6. Results

In this section, we present our experiments related to the recommendation quality of the similarity functions and the versions of feedback and content similarity. Our new methods are FC, Fisher conditional score from Section 3.5 followed by similarity, and FD, Fisher distance from Section 3.4 followed by similarity. We also investigate the size of the sample set used for defining these methods. As our final application of our feature representation, we experiment with using FC and FD as replacements for the neural embeddings in Gru4Rec described in Section 4.

### 6.1. Sample Set

**Figure 7:**
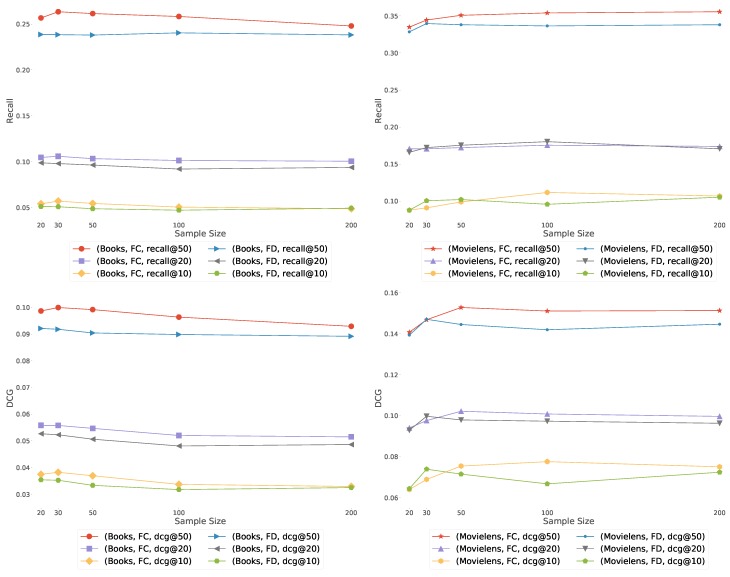
The quality of algorithms FD and FC with Jaccard similarity, as the function of the number of most popular items used as reference in the similarity graphs of Figure 1, Figure 2 and Figure 3 (horizontal axis). The Recall (**top**) and DCG (**bottom**) increases as we add more items in the sample set (i.e., list of recommended items).

The similarity graphs are defined via the set of items used as samples (Figure 1, Figure 2 and Figure 3). To smooth the Fisher vector representation of sparse items, we choose the most popular items in the training set as elements for the sample set. As we can see in Figure 7, recommendation quality saturates at a certain sample set size. Therefore, we set the size of the sample set to 10 or 20 for the remaining experiments.

### 6.2. Modalities: Implicit Feedback and Content

In Table 6 we show our experiments with DBPedia content as a modality on MovieLens. The overall best performing model is the multimodal Fisher with Jaccard similarity, while every unimodal Fisher method outperforms the baselines. By using Equation (37), we can blend different modalities, such as content and feedback, without the need of setting external parameters or applying learning for blending. We use a sample size of 10 in these experiments.

### 6.3. Recurrent Neural Networks and Fisher Embedding

**Figure 8:**
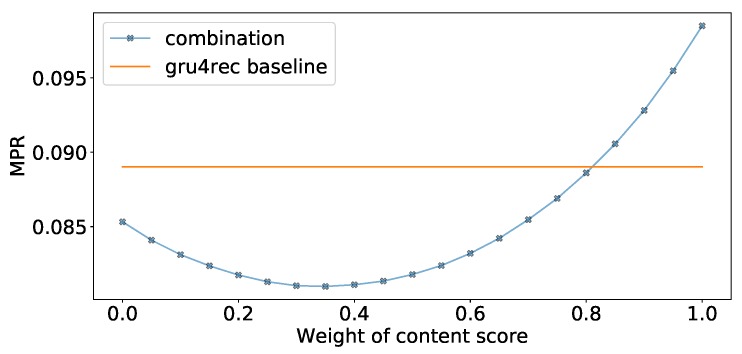
Linear combination weights for Feedback Jaccard and content based Fisher embedding models.

**Figure 9:**
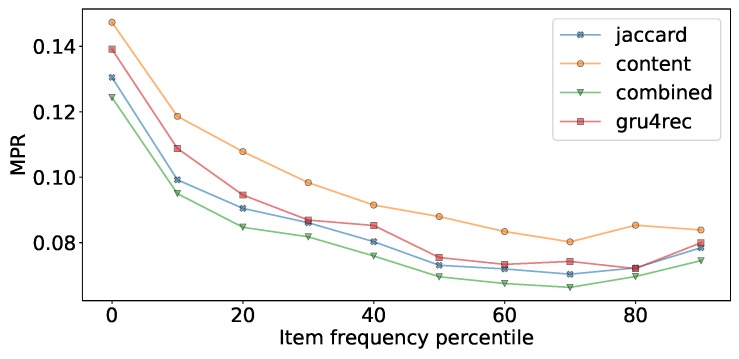
Performance of the different Gru4Rec based models in case of different item supports.

In Table 7 we show our experiments comparing the usage of Fisher embeddings with dynamically learned neural embeddings. The Fisher embedding based experiments are comparable to the baseline Gru4Rec even with simple similarity measures. In the last row of the table, we linearly combine the predicted scores of separately trained Gru4Rec networks using the feedback Jaccard similarity based Fisher embedding and the Content similarity based Fisher embedding. The effect of the linear combination is presented in Figure 8. The performance of the models in case of different item supports is presented in Figure 9.

We can observe that the performance of the feedback Jaccard similarity based Fisher embedding in combination with the Gru4Rec network performs similar to the dynamically learned neural embeddings of the original model, with the former performing better in terms of MPR and Recall, and the latter performing better in terms of DCG. While using content based Fisher embeddings by themselves produces worse results, these content based features combine well with the collaborative feedback-based ones. The good DCG performance of the original Gru4Rec model leads us to believe that this model places more emphasis on the top of the ranking list, in comparison to the Fisher embedding based versions, which perform better when measured by metrics that do not weigh by rank.

We also run experiments to measure the dependence of Gru4Rec performance on the input embeddings. We trained Gru4Rec by using randomly sampled vectors as input embeddings, and without any further modification of these vectors, the model still achieved an MPR score of 0.1642. While the score we achieved is weak compared to our other expriements, is still much better than the expected score of random item ordering. We conclude that the model is still able to learn the distribution of the items and certain item transitions via training its softmax layer.

### 6.4. Item-to-Item Methods over Infrequent Items

**Figure 10:**
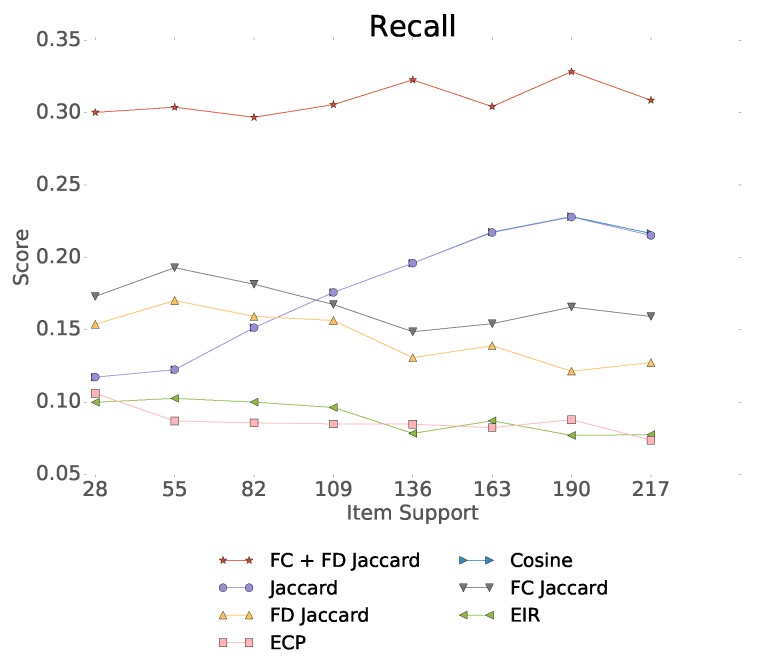
Recall@20 as the function of item support for the Netflix data set.

One of the main challenges in the field of recommendation systems is the “cold start” problem when the new items have too few transactions that can be used for modeling. Due to the importance of cold start recommendation, we examine the performance of our methods in the case of item transitions where the *next item* has low support. Figure 10 shows the advantage of the Fisher methods for item-to-item recommendation for different item support values. As support increases, best results are reached by blending based on item support. If the current session ends with an item of high support, we can take a robust baseline recommender, and if the support is less than roughly 100, we can use the FC or FD representation for constructing the recommendation.

Table 8, Table 9, Table 10 and Table 11 present our detailed results for item-to-item recommendation by using feedback similarity. The choice of the distance function strongly affects the performance of the Fisher models. As seen in Table 8, the overall best performing distance measure is Jaccard for both types of Fisher models. The results in Table 9, Table 10 and Table 11 show that the linear combination of the standard normalized scores of the Fisher methods outperforms the best unimodal methods (Fisher with Jaccard) for Netflix and Books, while for MovieLens and Yahoo! Music, Fisher distance with Jaccard performs best.

## 7. Conclusions

Recommending infrequent item-to-item transitions without personalized user history is a challenging dimensionality reduction task. In this paper, we considered the session-based item-to-item recommendation task, in which the recommender system has no personalized knowledge of the user beyond the last items visited in the current user session, a scenario that often occurs when physical sensors log the behavior of visitors indoors or outdoors.

We proposed Fisher information-based global item-item similarity models for the session modeling task. We reached improvement over existing methods in case of item-to-item transitions and session-based recommendations by experimenting with a variety of data sets as well as evaluation metrics. We constrained our similarity graphs for simple item-to-item transitions, defining the next item depending only on the last seen item. By using recurrent neural networks, we were able to expand our model to utilize more than one of the previous items in a session.

As a key feature, the model is capable of fusing different modalities, including collaborative filtering, content, and side information, without the need for learning weight parameters or using wrapper methods.

## Figures and Tables

**Table 1:** Data sets used in the experiments.

Data Set	Items	Users	Training Pairs	Testing Pairs
Netflix	17,749	478,488	7,082,109	127,756
MovieLens	3683	6040	670,220	15,425
Yahoo! Music	433,903	497,881	27,629,731	351,344
Books	340,536	103,723	1,017,118	37,403

**Table 2:** Co-occurrence quartiles.

Data Set	25%	50%	75%	Max
Books	1	1	2	1931
MovieLens	29	107	300	2941
Netflix	56	217	1241	144,817
Yahoo! Music	4	9	23	160,514

**Table 3:** Percentiles for the distribution of how many times a property is used in the knowledge graph. 75% of the properties are used only 42 times. We discard rare movie attributes, and only focus on Starring, writer, genre, director, and producer.

Mean	Std.	Min.	25%	50%	75%	Max.
1 K	5.3 K	1	1	3	42	70 K

**Table 4:** Top 5 movie features for the selected properties in the knowledge graph.

Property	Popular Values
Starring	Robin_Williams, Robert_De_Niro, Demi_Moore, Whoopi_Goldberg, and Bruce_Willis.
Writer	Woody_Allen, John_Hughes_(filmmaker), Robert_Towne, Lowell_Ganz, and Ronald_Bass.
Genre	Drama_film, Baroque_pop, Blues, Drama, and Rhythm_and_blues.
Director	Alfred_Hitchcock, Woody_Allen, Steven_Spielberg, Barry_Levinson, and Richard_Donner.
Producer	Walt_Disney, Arnon_Milchan, Brian_Grazer, Roger_Birnbaum, and Scott_Rudin.

**Table 5:** Statistics of the Jaccard similarity using the 100 most similar movies for each movie.

Mean	Std.	Min.	10%	20%	30.0%	40%	50%	60.0%	70%	80%	90%	Max.
0.1276	0.0728	0.0365	0.0778	0.087	0.0945	0.1005	0.1066	0.1179	0.126	0.1429	0.1976	0.8165

**Table 6:** Experiments on MovieLens with DBPedia content, all methods using Jaccard similarity.

	Recall@20	DCG@20
Collaborative baseline	0.139	0.057
Content baseline	0.131	0.056
FC content	0.239	0.108
FD content	0.214	0.093
FC multimodal	0.275	0.123

**Table 7:** Experiments on MovieLens with different input embeddings in Gru4Rec. Best performing methods are indicated in boldface.

	MPR	DCG@20	Recall@20
Random embedding	0.1642	0.296	0.582
Neural embedding	0.0890	**0.466**	0.799
Feedback Jaccard based Fisher embedding	0.0853	0.437	0.794
Content based Fisher embedding	0.0985	0.405	0.757
Feedback and Content combination	**0.0809**	0.446	**0.803**

**Table 8:** Experiments with combination of collaborative filtering for the first quantile (based on KDE estimation of 25%) of the MovieLens data. Best performing methods are indicated in boldface.

	MPR	Recall@20	DCG@20
Cosine	0.4978	0.0988	0.0553
Jaccard	0.4978	0.0988	0.0547
ECP	0.4976	0.0940	0.0601
EIR	0.3203	0.1291	0.0344
FC Cosine	0.3583	0.1020	0.0505
FD Cosine	0.2849	0.1578	0.0860
FC Jaccard	0.3354	0.1770	**0.1031**
FD Jaccard	**0.2415**	**0.1866**	0.1010
FC ECP	0.2504	0.0940	0.0444
FD ECP	0.4212	0.1626	0.0856
FC EIR	0.4125	0.0861	0.0434
FD EIR	0.4529	0.1068	0.0560

**Table 9:** Experiments over the first quantile (based on KDE estimations of 25%). Best performing methods are indicated in boldface.

		MovieLens	Goodreads	Yahoo! Music	Netflix
MPR	Cosine	0.5024	0.4995	0.5	0.5028
	Jaccard	0.5024	0.4995	0.5	0.5028
	ECP	0.4974	0.5004	0.4999	0.4968
	EIR	0.3279	0.482	0.2437	0.3395
	FC Jaccard	0.2665	0.3162	0.2456	0.4193
	FD Jaccard	**0.2382**	**0.2389**	**0.0564**	**0.307**
	FC + FD JC	0.3652	0.2751	0.1319	0.3792
Recall@20	Cosine	0.0988	0.0966	0.0801	0.1254
	Jaccard	0.0988	0.0966	0.0801	0.1254
	ECP	0.0893	0.0956	0.0801	0.0954
	EIR	0.1212	0.0996	0.1324	0.1033
	FC Jaccard	0.1834	0.1084	0.1358	0.1845
	FD Jaccard	**0.1866**	0.0917	**0.2334**	0.1636
	FC + FD JC	0.118	**0.136**	0.101	**0.3034**
DCG@20	Cosine	0.0518	0.0505	0.044	0.0739
	Jaccard	0.0518	0.0505	0.044	0.0733
	ECP	0.0528	0.0505	0.044	0.0772
	EIR	0.0405	0.0635	0.05	0.1198
	FC Jaccard	0.1045	0.0517	0.0663	0.106
	FD Jaccard	**0.1085**	0.0462	**0.1112**	0.0971
	FC + FD JC	0.071	**0.0757**	0.0559	**0.1734**

**Table 10:** Experiments over the first two quantiles (based on KDE estimations of 50%). Best performing methods are indicated in boldface.

		MovieLens	Goodreads	Yahoo! Music	Netflix
MPR	Cosine	0.5145	0.4995	0.5002	0.5017
	Jaccard	0.5143	0.4995	0.5002	0.5014
	ECP	0.4836	0.5004	0.4997	0.4953
	EIR	0.3474	0.482	0.2495	0.3522
	FC Jaccard	0.3181	0.3162	0.2452	0.4534
	FD Jaccard	**0.2589**	**0.2389**	**0.0629**	**0.3191**
	FC + FD JC	0.3167	0.2751	0.1357	0.3634
Recall@20	Cosine	0.1099	0.0966	0.0958	0.1792
	Jaccard	0.1099	0.0966	0.0958	0.1789
	ECP	0.1001	0.0956	0.0958	0.0863
	EIR	0.1066	0.0996	0.1109	0.0914
	FC Jaccard	0.137	0.1084	0.121	0.1683
	FD Jaccard	**0.1411**	0.0917	**0.208**	0.1448
	FC + FD JC	0.0981	**0.136**	0.1034	**0.3071**
DCG@20	Cosine	0.0572	0.0505	0.0532	0.0987
	Jaccard	0.0574	0.0505	0.0532	0.097
	ECP	0.0541	0.0505	0.0532	0.1104
	EIR	0.0474	0.0635	0.0459	0.1283
	FC Jaccard	0.0729	0.0517	0.0628	0.0973
	FD Jaccard	**0.0787**	0.0462	**0.1017**	0.0833
	FC + FD JC	0.0538	**0.0757**	0.0567	**0.1743**

**Table 11:** Experiments over the first three quantiles (based on KDE estimations of 75%). Best performing methods are indicated in boldface.

		MovieLens	Goodreads	Yahoo! Music	Netflix
MPR	Cosine	0.5223	0.4992	0.4989	0.4912
	Jaccard	0.5203	0.4992	0.4989	0.4865
	ECP	0.4668	0.5007	0.501	0.4866
	EIR	0.3578	0.4663	0.254	0.3775
	FC Jaccard	0.4406	0.3257	0.256	0.4491
	FD Jaccard	0.3987	**0.2502**	**0.0763**	**0.3441**
	FC + FD JC	**0.3431**	0.2871	0.1507	0.3613
Recall@20	Cosine	**0.1233**	0.0979	0.0958	0.1996
	Jaccard	0.1226	0.0979	0.0958	0.1588
	ECP	0.096	0.0961	0.0927	0.0689
	EIR	0.1052	0.1048	0.1206	0.0724
	FC Jaccard	0.1225	0.1182	0.1316	0.2023
	FD Jaccard	0.1133	0.0891	**0.2068**	0.0983
	FC + FD JC	0.0969	**0.1416**	0.1215	**0.3235**
DCG@20	Cosine	0.0655	0.0499	0.0528	0.1127
	Jaccard	0.0655	0.0499	0.0528	0.0913
	ECP	0.0588	0.0499	0.0528	0.1655
	EIR	0.0495	0.0584	0.0545	0.1382
	FC Jaccard	**0.0657**	0.0582	0.0681	0.114
	FD Jaccard	0.0587	0.0467	**0.1044**	0.0542
	FC + FD JC	0.0506	**0.0822**	0.0686	**0.1827**

## Abbreviations

The following abbreviations are used in this manuscript:

DCG	Discounted Cumulative Gain [55]
ECP	Empirical Conditional Probability
EIR	Euclidean Item Recommender [6]
FC	Model from Section 3.5 followed by similarity
FD	Model from Section 3.4 followed by similarity
GRU	Gated Recurrent Unit [17]
Gru4Rec	Recommender algorithm using GRU units [7]
KDE	Kernel Density Estimation
RNN	Recurrent Neural Network
